# The health case for urgent action on climate change

**DOI:** 10.1136/bmj.m1103

**Published:** 2020-03-30

**Authors:** Andy Haines, Pauline Scheelbeek, Kamran Abbasi

**Affiliations:** 1 Centre on Climate Change and Planetary Health, London School of Hygiene and Tropical Medicine, London, UK; 2 The BMJ, London, UK

## Abstract

Health professionals have a leading role

It is about 30 years since warnings first appeared in prominent journals about the potential for large and wide ranging effects on human health from climate and other global environmental changes.^1^ ^2^ ^3^ To date, global action to tackle these burgeoning threats remains inadequate both in scale and in speed. For example, the pledged nationally determined contributions to reducing greenhouse gas emissions—as enshrined in the 2015 Paris Agreement on Climate Change—are a first step, but even if fully implemented (and this is by no means certain), global average temperatures are still likely to be more than 3°C above pre-industrial levels by the end of the century.^4^ With higher levels of heating expected over land mass than over the oceans, accompanied by changes in rainfall patterns, the long term consequences for human health are far reaching.^5^

## Safeguarding human health and livelihoods

The need to protect critically important natural systems is clear. Many of the services provided by nature are important for safeguarding human health and livelihoods. These include the provision of clean water and air, pollination of crops, and medicinal products. The 2019 Intergovernmental Science-Policy Platform on Biodiversity and Ecosystem Services report showed that about 1 million species face extinction without rapid interventions to tackle the drivers of biodiversity loss, such as land use and climate change.^6^ The covid-19 pandemic reminds us that many emerging diseases arise from complex interactions between humans, wildlife, and domestic animals, resulting from changes in land use or food systems.^7^ Marine and terrestrial ecosystems also sequester carbon from the atmosphere and therefore have a vital role in climate mitigation efforts.^8^

The rapid pace of environmental change, especially over recent years, has led to the definition of a new geological epoch, widely known as the Anthropocene, and characterised by the dominance of human activities on Earth systems.^9^

In early 2019, *The BMJ* issued a call for papers that dealt with the challenges of the Anthropocene for human health and identified opportunities for action.^10^ For example, phasing out fossil fuels could yield major near term health benefits from reduced air pollution, potentially averting millions of premature deaths annually,^11^ as well as reducing greenhouse gas emissions and thus the risks of dangerous climate change. In recent months, several research papers submitted in response to the call have appeared in *The BMJ* and have discussed the effects on health of predominantly anthropogenic pollutants—including ozone and fine particulate air pollution.^12^ ^13^ ^14^ We are now fully launching the series by publishing several more articles that emphasise potential ways to reduce the environmental footprint of society and improve public health (https://www.bmj.com/anthropocene), with more to follow in the next few months. These will include papers on decarbonising the NHS; capitalising on the health (co)benefits of “low carbon” policies in sectors such as energy, housing, transport, and food systems; and the health dividend that could be achieved through more sustainable cities.

## Zero emissions target

The UK parliament has declared a climate emergency, and the Committee on Climate Change recommends a target of net zero emissions by 2050,^15^ but it remains unclear how complete decarbonisation can be achieved in practice. Furthermore, it remains uncertain how “emission leakage” (such as replacing UK goods and services with carbon intensive alternatives from countries with lower ambitions for emission reduction) can be avoided. On a global scale the challenges are deeply concerning, with the US planning to leave the Paris agreement and many countries seeming reluctant to act decisively and expeditiously.

On biodiversity loss and other issues that typically receive less attention, political will also is lacking for timely action at scale. For these reasons health professionals have a responsibility to act locally, nationally, and internationally—both as individuals and through their professional bodies—taking a leading role in supporting the implementation of policies that will protect health and tackle the pressing challenges of the Anthropocene. With health services accountable for about 4.4% of total greenhouse gas emissions globally (6.3% for the NHS and Public Health England in the UK),^16^ we clearly need simultaneous transformational change in other sectors—such as energy, food, housing and transport—to make a meaningful contribution to protecting health. This will make the leading role of health professionals in tackling these issues even more pivotal for public health impact.

We hope that this series will provoke debate and discussion, and stimulate more inquiry in this critical area, especially with a focus on solutions. However, there is much more to be done. In November this year the 26th Conference of the Parties (COP) is scheduled to be hosted in Glasgow.^17^ This will be an important opportunity to raise the profile of the interlinkages between climate change and health, and stress the opportunities—in various sectors—to contribute to protecting health in the Anthropocene.

## Notes

Provenance and peer review: Commissioned; externally peer reviewed.

This article is part of a series commissioned by *The BMJ*. Open access fees are paid by the Wellcome Trust, which had no involvement in external peer review, editing, or the decision to publish.
